# A Novel miRNA From Egg-Derived Exosomes of *Schistosoma japonicum* Promotes Liver Fibrosis in Murine Schistosomiasis

**DOI:** 10.3389/fimmu.2022.860807

**Published:** 2022-04-27

**Authors:** Yiluo Wang, Wenci Gong, Hao Zhou, Yuan Hu, Lan Wang, Yujuan Shen, Guoying Yu, Jianping Cao

**Affiliations:** ^1^National Institute of Parasitic Diseases, Chinese Center for Disease Control and Prevention (Chinese Center for Tropical Diseases Research), Key Laboratory of Parasite and Vector Biology, National Health Commission of the People’s Republic of China, World Health Organization Collaborating Center for Tropical Diseases, Shanghai, China; ^2^State Key Laboratory of Cell Differentiation and Regulation, College of Life Science, Henan Normal University, Xinxiang, China; ^3^The School of Global Health, Chinese Center for Tropical Diseases Research, Shanghai Jiao Tong University School of Medicine, Shanghai, China

**Keywords:** *Schistosoma japonicum*, schistosomiasis, exosomes, microRNAs, hepatic fibrosis, TGF-β pathway, TGF-β RI

## Abstract

Schistosomiasis caused by *Schistosoma japonicum* is a serious public health problem in China. Granuloma and hepatic fibrosis are the main pathological features of schistosomiasis japonica. The role and mechanism of egg-derived exosomes of *S. japonicum* in liver fibrosis remain unclear. In this study, we found that egg-derived exosomes of *S. japonicum* carry a new type of microRNA (miRNA-33). *In vitro*, this novel miRNA upregulated the expression of smooth muscle actin (α-SMA) and collagen 1 α1 (Col 1 α1) in the human hepatic stellate cell (LX-2) line at both mRNA and protein levels. *In vivo*, this novel miRNA was upregulated in the serum of infected mice, and when injected into mice through the tail vein using miRNA agomir, α-SMA, Col 1 α1, and Col 3 α1 were upregulated in liver tissue at both mRNA and protein levels. In addition, this novel miRNA downregulated the expression of α-SMA and Col 1 α1 in liver tissue at mRNA and protein levels in mice infected with *S. japonicum* and treated with miRNA antagomir. The novel miRNA-33 upregulated TGF-β Receptor I (TGF-β RI) at both mRNA and protein levels in LX-2 cells. Our results suggest that this novel miRNA from egg-derived exosomes of *S. japonicum* can promote liver fibrosis in the host in a cross-species manner, and the degree of fibrosis can be decreased by inhibiting the expression of this miRNA.

## Introduction

Schistosomiasis is a zoonotic parasitic disease that seriously endangers human health. The most prevalent forms of schistosomiasis diseases worldwide are caused by three main species that infect human: *Schistosoma haematobium*, *S. japonicum*, and *S. mansoni*. Approximately 200 million people are infected every year, and 800 million people are at risk of infection worldwide (1).

Hepatic fibrosis is characterized by the accumulation of extracellular matrix (ECM), which is mainly composed of type I collagen, and activated hepatic stellate cells (HSCs). In fibrotic liver, quiescent HSCs transdifferentiate into proliferative, migratory, and contractile myofibroblasts, resulting in pro-fibrotic transcriptional and secretory properties, and secretion of ECM molecules that accumulate in the intercellular space, leading to fibrosis (2).

According to previous studies, eggs in the host liver are surrounded by immune cells, forming granulomas (3). It is well established that *S. japonicum* infection causes granulomatous responses to parasite eggs trapped in the liver, resulting in severe liver fibrosis and circulatory impairment, and eventually loss of the ability to work and self-care, and even death (4). Additionally, schistosome egg-triggered granuloma and pipestem fibrosis can occur within liver sinusoids and branches of the portal vein (5). Egg-derived exosomes of *S. japonicum* have attracted significant research interest, and they can be delivered to other cells through extracellular vesicles to modulate the gene expression and phenotype of distant recipient cells (6). MicroRNAs (miRNAs) are a class of endogenous non-coding single-stranded RNA molecules 20-24 base pairs (bp) in length that are evolutionarily conserved and play a key role in regulating gene expression. They can specifically and effectively inhibit antisense oligonucleotides by chemical modification, and mRNA targets can also regulate the levels and functions of miRNAs (7, 8). The effects of miRNAs have demonstrated that activation and transformation of HSCs are crucial for the pathogenesis of liver fibrosis (9).

In the present study, we identified and characterized a novel miRNA (novel miRNA-33) from *S. japonicum*, which can activate HSCs of parasite-infected mice, leading to liver fibrosis. More importantly, the degree of liver fibrosis in infected mice was reduced after treatment with miRNA antagomir. Our results suggest that novel miRNA-33 promotes fibrosis by targeting TGF-β receptor I (TGF-β RI).

## Materials and Methods

### Ethics Statement

All animal experiments were performed in strict accordance with the Regulations for the Administration of Affairs Concerning Experimental Animals (approved by the State Council of People’s Republic of China), and efforts were made to minimize suffering. The study was approved by the Laboratory Animal Welfare & Ethics Committee (LAWEC) of the National Institute of Parasitic Diseases, Chinese Center for Disease Control and Prevention (Chinese Center for Tropical Diseases Research; approval ID: IPD 2019–12).

### Infection of Laboratory Animals

Thirty-six 6-week-old female C57BL/6 mice for establishment of a fibrosis mouse model were divided into negative control (NC), novel 33 agomir, and normal saline groups. In the agomir NC group, nine mice were injected with 120 μL of 20 nM agomir NC (Ribo, China) *via* the tail vein once a week for 8 weeks. In the novel 33 agomir group, 18 mice were injected with 120 μL of 20 nM agomir novel 33 (Ribo) *via* the tail vein once a week for 8 weeks. In the normal saline group, nine mice were injected with 120 μL of normal saline *via* the tail vein once a week for 8 weeks. MiRNA agomir NC and miRNA agomir novel 33 were dissolved in sterile normal saline. C57BL/6 mice were euthanized after 6 weeks with miRNA agomir, and their livers were removed for subsequent experimental studies.

Forty-five 6-week-old female C57BL/6 mice for establishment of an *S. japonicum* infection and treatment model were divided into NC, infected treatment, infection, and normal groups. In the antagomir NC group, nine mice were percutaneously exposed to 20 ± 1 *S. japonicum* cercariae, and 120 μL of 20 nM miRNA antagomir NC (Ribo) was injected *via* the tail vein 7 days after mice were infected with *S. japonicum* cercariae, once a week for 8 weeks. In the infection treatment group, 18 mice were percutaneously exposed to 20 ± 1 *S. japonicum* cercariae, and 120 μL of 20 nM miRNA antagomir novel 33 (Ribo) was injected *via* the tail vein 7 days after the mice were infected with *S. japonicum* cercariae, once a week for 8 weeks. In the infection group, nine mice were percutaneously exposed to 20 ± 1 *S. japonicum* cercariae, and 120 μL of normal saline was injected *via* the tail vein 7 days after the mice were infected with *S. japonicum* cercariae, once a week for 8 weeks. In the normal group, the nine mice were not treated. MiRNA antagomir NC and miRNA antagomir novel 33 were dissolved in sterile normal saline. Six weeks after C57BL/6 mice were infected with *S. japonicum* and cured with miRNA antagomir, they were euthanized and their livers were removed for subsequent experimental studies.

In order to obtain a large number of *S. japonicum* eggs, 2.5 kg healthy female white rabbits were percutaneously exposed to 800 ± 20 *S. japonicum* cercariae. After 6 weeks of infection, rabbits were euthanized, livers were harvested, and fresh *S. japonicum* eggs were collected.

The above 6-week-old female C57BL/6 mice were purchased from Shanghai Ji-hui Experimental Animal Co., Ltd, and raised in the specific pathogen-free (SPF) animal room of the National Institute of Parasitic diseases, Chinese Center for Disease Control and prevention (National Tropical Disease Research Center). Female white rabbits were purchased from Shanghai Song-lian Experimental Animals Co., Ltd. (Shanghai, China). *S. japonicum* cercariae were provided by the National Institute of Parasitic Diseases, Chinese Center for Disease Control and Prevention (National Tropical Disease Research Center).

### Egg Counting in Livers of Mice Infected With *S. japonicum*

The mouse liver worm technique was based on previous studies (10) with some modifications. Six weeks after mice were infected with *S. japonicum* cercariae and cured with miRNA antagomir, mouse livers were removed, weighed, 5 g of liver tissue was chopped into small pieces, fragments were placed in 15 mL of 1% sodium hydroxide solution, and digested at 37°C for 2 h until fragments had completely disappeared. After digestion, 10 μL of the digestion solution containing *S. japonicum* eggs was placed under a microscope, eggs were counted, and the liver group of each mouse was independently counted three times.

### Acquisition of Egg-Derived Exosomes and Exosome-Derived miRNAs of *S. japonicum*

*S. japonicum* eggs were extracted as described previously (11) with some modifications. After 6 weeks of infection, livers of female white rabbits were ground into a homogenate using a high-speed tissue grinder. The homogenate was diluted with cold phosphate-buffered saline (PBS) containing 2% penicillin and streptomycin, then filtered successively through 80-mesh and 100-mesh screens. The filtered homogenate was transferred to a 50 mL centrifuge tube. The tube was centrifuged at 100 × *g* for 5 min at 4°C, and the supernatant was discarded. Centrifugation was repeated several times until the supernatant was clear. The pellet was digested in 1 mg/mL collagenase IV (2091MG100, BioFroxx, Germany) for 1.5 h. After washing with cold PBS, eggs were resuspended in Roswell Park Memorial Institute (RPMI)-1640 medium (Thermo Fisher Scientific, USA). Eggs were then cultured in RPMI-1640 medium at 37°C with 5% CO_2_. The egg culture supernatant was sampled every 24 h, and stored at -80°C. Supernatant samples were ultra-centrifuged as described previously (12, 13), resulting in sterile egg-derived exosomes of *S. japonicum*.

### TEM Negative Staining

Sterile egg-derived exosomes of *S. japonicam* (20 μL) were resuspended in 100 μL of PBS, placed on a 150-mesh carbon film copper mesh for 1 min, and excess liquid was wiped off using filter paper. Grids were negatively stained with 2% phosphor-tungstic acid (G1102, Servicebio, China) for 1 min and dried at room temperature (RT). Grids were loaded onto the sample holder of the transmission electron microscope (HITACHI, Tokyo, Japan) and exposed at 80 kV for image capture.

### Cell Culture and Transfection

Human HSCs (LX-2, provided by Wuxi Newgain Biotechnology Co., Ltd., China) were grown in Dulbecco’s modified Eagle’s medium (DMEM; Thermo Fisher Scientific) supplemented with 10% heat-inactivated fetal bovine serum (FBS; Thermo Fisher Scientific), 100 U/mL penicillin, and 100 µg/mL streptomycin (Thermo Fisher Scientific) at 37°C with 5% CO_2_. When cells had reached a density of 1×10^6^ per well in the 6-well plate, they were transfected with 80 nM novel miRNA-33 mimic (Ribo) or corresponding NC mimic (Ribo), or LX-2 cells were stimulated with 120 μL of egg-derived exosomes of *S. japonicum* at a protein concentration of 65 μg/mL. The detailed sequences of miRNA mimics are shown in **Table 1**.

**Table 1:** miRNA sequences.

Gene	Sequences
U6 (human/mouse)	GUGCUCGCUUCGGCAGCACAUAUA
	CUAAAAUUGGAACGAUACAGAGAA
	GAUUAGCAUGGCCCCUGCGCAAGG
	AUGACACGCAAAUUCGUGAAGCGU
	UCCAUAUUUUU
	
Novel miRNA-33	GAGUGCAGUUGAAGUGGCU
Novel miRNA-68	AUCUCGGUCGUUGUGGUGACUU
miR-1	UGGAAUGUGGCGAAGUAUGGUC
Novel miRNA-30	AAGAGAGGCUGUAUUGAACC

### Histological Evaluation

Fresh liver tissues were fixed in 4% formaldehyde overnight and paraffin-embedded using routine procedures. Paraffin sections (5 μm) were prepared from each liver tissue sample. Liver tissue sections were stained with Masson’s trichrome staining to evaluate collagen content and distribution. Collagen fibers were stained blue, cell nuclei were stained black, and the background was stained red. Each stained section was examined by optical microscopy at 100 × magnification with identical settings. Thirty images of granulomas around single eggs were captured from three sections in each tissue, and every picture was evaluated in a double-blind fashion by two independent investigators. The areas featuring granulomas and fibrosis surrounding single eggs were evaluated using image J (NIH, USA). The positive rate of collagen was calculated as the collagen area of the selected area divided by the total area of the selected area.

In order to further observe the degree of liver fibrosis, immunofluorescence experiments were performed as previously described (14) with some modifications. Paraffin sections were dewaxed and washed successively, then incubated with 0.25% pepsin at 37°C for 20 min, and distilled water as added to prevent the tissue from drying. The antigen was repaired, washed, and dried. A circle was drawn around the tissue with a histochemical pen and sealed with bovine serum albumin (BSA) for 30 min. After gentle shaking in blocking solution, primary antibody in PBS was placed on slices, and incubated at 4°C overnight. After washing, slides were incubated with secondary antibody covering the circle at room temperature in the dark for 50 min. After washing, autofluorescence quenching agent was added to the circle for 5 min, then rinsed with running water for 20 min. After washing, slices were washed with PBS, dried, and 4’,6-diamidino-2-phenylindole (DAPI) dye was added in the circle and incubated at room temperature without light for 10 min. Slices were then placed in PBS and washed by shaking on a decolorization shaking table. After slices had dried slightly, they were sealed with anti-fluorescence quenching sealing agent. After sealing, slices were observed under a fluorescence microscope and images were captured. Antibody information is listed in **Table 2**.

**Table 2:** Information for immunofluorescence antibodies and other reagents.

Reagent	Manufacturer	Cat. No.	Dilution ratio
BSA	Servicebio		
α-SMA Col 1 (α1)	CST Servicebio	#19245 GB11022-3	1:400 1:100
Col 3 (α1) 488-Goat anti-rabbit DAPI	Servicebio Servicebio Servicebio	GB13023-2 GB25303 G1012	1:400 1:400
Antifade-mounting medium	Servicebio	G1401	

### Quantitative Real-Time Reverse Transcription PCR (qPCR)

TRIzol reagent (Sigma Aldrich, USA) was used to extract total RNA from liver tissues of miRNA-induced fibrosis and *S. japonicum* infection and treatment groups, and from human LX-2 HSCs after transfection (15), and detection was carried out according to the manufacturer’s instructions. Reverse-transcription was performed using a cDNA reverse transcription kit (Cat# R202-02, EnzyArtisan Biotech, China). The resulting cDNA was used as template for qPCR with SYBR Green Real-time PCR Master Mix (Cat# Q204-01, EnzyArtisan Biotech) and 0.4 μM forward and reverse primers. All miRNAs in serum from C57BL/6 mice were extracted using miRNeasy Serum/Plasma Kit (Cat. No. 217184, Qiagen, Germany) according to the manufacturer’s recommendations, and all miRNAs from liver tissue of C57BL/6 mice were extracted following the instructions supplied with the TRIzol Reagent Kit (Invitrogen, USA). All the miRNA reverse transcription operations mentioned above were carried out according to the guidance of EnzyArtisan Biotech (Cat# R601, EnzyArtisan Biotech). The U6 gene was used in qPCR experiments on novel miRNA 33 as an internal reference for miRNA, and glyceraldehyde-3-phosphate dehydrogenase (GAPDH) served as an endogenous control to normalize mRNA levels. Relative expression levels of *Col 1 (α1), Col 3 (α1)*, and *α-SMA* were calculated by the 2^- ΔΔ CT^ method. The primers used in this study are listed in **Table 3**.

**Table 3:** Primer sequences.

Gene	Primer sequences
Human GAPDH-F	GTCTCCTCTGACTTCAACAGCG
Human GAPDH-R	ACCACCCTGTTGCTGTAGCCAA
Human smooth muscle actin (α-SMA) (ACTA2)-F	ATGCTTCTAAAACACTTTCCTGCTC
Human smooth muscle actin (α-SMA) (ACTA2)-R	AGCTTTGGCTAGGAATGATTTGG
Human Col 1 (α1)-F	GGTTCGGAGGAGAGTCAGGAAG
Human Col 1 (α1)-R	TTTCAGCAACACAGTTACACAAGG
Human Col 3 (α1) -F	TGGTCTGCAAGGAATGCCTGGA
Human Col 3 (α1) -R	TCTTTCCCTGGGACACCATCAG
Human TGF-β RI-F	GACAACGTCAGGTTCTGGCTCA
Human TGF-β RI-R	CCGCCACTTTCCTCTCCAAACT
	
Mouse GAPDH-F	CATCACTGCCACCCAGAAGACTG
Mouse GAPDH-R	ATGCCAGTGAGCTTCCCGTTCAG
Mouse smooth muscle actin (α-SMA) (ACTA2)-F	TGCTGACAGAGGCACCACTGAA
Mouse smooth muscle actin (α-SMA) (ACTA2)-R	CAGTTGTACGTCCAGAGGCATAG
Mouse Col 1 (α1)-F	CCTCAGGGTATTGCTGGACAAC
Mouse Col 1 (α1)-R	CAGAAGGACCTTGTTTGCCAGG
Mouse Col 3 (α1)-F	GACCAAAAGGTGATGCTGGACAG
Mouse Col 3 (α1)-R	CAAGACCTCGTGCTCCAGTTAG
	
Novel miRNA-33 reverse (for RT PCR)	GTCGTATCCAGTGCAGGGTCCGAG
	GTATTCGCACTGGATACGACAGCC
	AC
Novel miRNA-33 qPCR-F	GCGCGGAGTGCAGTTGAA
Novel miRNA-33 qPCR-R	AGTGCAGGGTCCGAGGTATT
U6 reverse (for RT PCR)	GTCGTATCCAGTGCAGGGTCCGAG
	GTATTCGCACTGGATACGACAAAA
	ATATGG
U6 qPCR-F	GCTCGCTTCGGCAGCACATATAC
U6 qPCR-R	AGTGCAGGGTCCGAGGTATT

### Western Blotting

Proteins were extracted from liver tissues of miRNA-induced fibrosis and *S. japonicum* infection and treatment groups, and from human LX-2 HSCs after transfection as described previously with some modifications (16). Samples of LX-2 cells or fresh liver tissue from infected mice were washed three times with cold PBS. To prepare protein samples, phosphatase and protease inhibitor were used during lysis of mouse tissue and LX-2 cell samples. Tissue and cell samples were lysed at 4°C for 20 min, centrifuged at 10, 000 × *g* for 15 min at 4°C, and the protein concentration of supernatants was determined by the BCA method (T9300A-1, TaKaRa, Japan). An aliquot of supernatant was mixed with 6 × protein loading buffer (DL101-02, TransGen Biotech, China) and denatured at 100°C for 15 min. The protein concentration of fresh exosomes extracted as described above was determined according to the BCA protein determination method (TaKaRa). 200 μL suspension of exosomes of *S. japonicum* was mixed with 40 μL of 6 × protein loading buffer (DL101-02), and incubated in a metal bath at 100°C for 10 min. To each lane was added 20 μg total protein and proteins were separated by 10% sodium dodecyl sulfate polyacrylamide gel electrophoresis (SDS-PAGE, P0012A, Beyotime, China). After electrophoresis, proteins were transferred to a polyvinylidene fluoride (PVDF) membrane (0.22 μm) according to the manufacturer’s semi dry transfer method (Bio-Rad, USA). Next, the PVDF membrane was blocked with protein-free fast blocking solution (PS108, Shanghai Epizyme Biomedical Technology Co., Ltd, China) under shaking at room temperature for 20 min. Tris-buffered saline (TBS) containing 0.5‰ Tween-20 was used to wash the PVDF membrane three times for 5 min each time. Primary antibodies were anti-TGF-β RI (bs-0638R, Bioss, China), anti-GAPDH (5174S, Cell Signaling Technology, CST, USA), anti-α-SMA (19245S, CST), anti-Col 1 α1 (bs-7158R, Bioss), anti-Col 3 α1 (22734-1-AP, Proteintech, USA), anti-Smad3 (9523S, CST), anti-phospho-Smad3 (9520S, CST), anti-Flotillin-1 (3253S, CST), and anti-HSP70 (46477S, CST). PVDF membranes were incubated at 4°C overnight, then incubated with secondary antibody at room temperature for 1.5 h after washing three times with TBST. Anti-rabbit IgG, a horseradish peroxidase (HRP)-conjugated antibody (7074S, CST) was used as secondary antibody, along with anti-mouse IgG, also an HRP-conjugated antibody (7076S, CST). Membranes were washed three times with TBS containing 0.5‰ Tween-20 for 5 min each time under sealed conditions. Immunoreactive bands were visualized on digital images captured with a ChemiDoc MP Imaging System (Bio-Rad) using Tanon High-sig ECL Western Blotting Substrate (180-501, Tanon, China). After visualization, the band intensities were quantified by ImageJ software (17, 18).

### Construction of a 3’-Untranslated Region (UTR) Luciferase Reporter Gene

According to miRDB online bioinformatics analysis software (19, 20), the seed sequence of novel miRNA-33 was predicted to complement the 3’UTR of TGF-β RI. In order to detect whether TGF-β RI was a target of novel miRNA-33, wild-type (WT) and mutant 3’UTRs of TGF-β RI were synthesized by chemical synthesis according to the 3’UTR sequence information and the specific TGF-β RI mutation site, and cloned into the pmiR-RB-REPORT (pmirGLO) dual luciferase reporter vector. At the logarithmic growth stage, 293T cells were inoculated into 96-well plates at a cell density of 1.5 × 10^4^ cells per well and cultured in a 37°C incubator for 24 h, 10 μL Opti-MEM medium (Thermo Fisher Scientific) was used to dilute miRNA mimics or NC mimic, 15 μL of target gene 3’UTR double reporter gene vector or mutant vector in Opti-mem medium, or 25 μL of Opti-mem medium containing 0.25 μL of Lipofectamine 2000 reagent were added and incubated for 5 min with gentle shaking. After standing for 20 min, 50 μL was removed and made up to 100 μL with buffer. The transfection concentration of mimics was 50 nM and the plasmid concentration was 250 ng/well. Three wells were included for each group. After 6 h, 100 μL of fresh medium was added. At 48 h after transfection, medium was dispensed at 35 μL/well, luciferase substrate in 1 × PBS was added at 35 μL/well, incubated with shaking for 10 min, and fluorescence was measured after adding 30 μL of stop reagent and incubating with shaking for 10min. The reporter fluorescence of the vector used was hLuc, and the calibration fluorescence was hRLuc (internal reference correction). The fluorescence value was measured using a fluorescence illuminometer (21).

### Statistical Analyses

All data are presented as means ± standard deviation (SD), all results were analyzed using GraphPad Prism 6.0 software (22). Student’s *t*-tests were applied to assess differences between two groups, one-way analysis of variance (ANOVA) followed by Tukey’s post-tests were used for three or more groups, and *p <*0.05 was considered to indicate statistical significance.

## Results

### Identification of Egg-Derived Exosomes of *S. japonicum*

**Figure 1:**
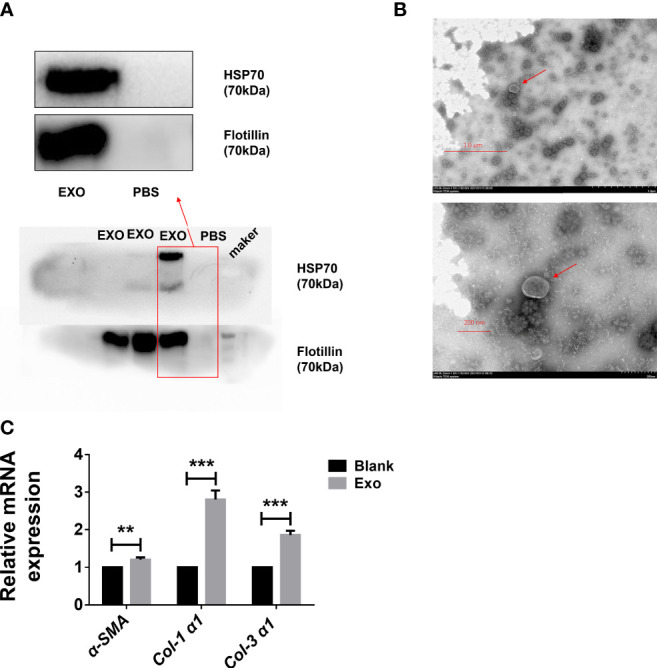
Egg-derived exosomes from *Schistosoma japonicum* can promote liver fibrosis. **(A)** Western blotting detection of markers of egg-derived exosomes of *S. japonicum*. **(B)** Transmission electron microscopy (TEM) analysis of exocrine. **(C)** At 24 h after stimulation of LX-2 human hepatic stellate) cells (HSCs) by egg-derived exosomes of *S. japonicum*, mRNA levels of *smooth muscle actin (α-SMA), collagen (Col) 1 (α1)*, and *collagen (Col) 3 (α1)* were measured by qPCR. Results are averaged from three independent experiments, and Student’s *t*-tests were applied to assess differences between two groups. The mRNA expression levels of *α-SMA*, *Col 1 (α1)* and *Col 3 (α1)* were normalized against *GAPDH* **p < 0.001, ***p < 0.0005.

In order to identify egg-derived exosomes of S. *japonicum*, we referred to a previously reported exosomes marker protein to preliminarily identify egg-derived exosomes of *S. japonicum*. Specifically, we identified the obtained egg-derived exosomes of *S. japonicum* by immunoblotting and transmission electron microscopy. The results showed that the egg-derived exosomes of *S. japonicum* contained flotillin-1, HSP-70, consistent with previous reports (23, 24)(**Figure 1A**). Furthermore, to verify the purity and quality of the exosomes, negative-staining transmission electron microscopy (TEM) was conducted to evaluate exosome morphology. The results showed that our previously obtained samples resembled exosomes (**Figure 1B**). This suggests that we successfully obtained fresh egg-derived exosomes of *S. japonicum*.

### Egg-Derived Exosomes of *S. japonicum* Can Activate LX-2

Although we identified egg-derived exosomes of *S. japonicum*, their role in liver fibrosis remains unclear. We carried out *in vitro* experiments to test whether exosomes can activate hepatic stellate cells. We stimulated LX-2 cells with 120 μL of 65 μg/mL egg-derived exosomes of *S. japonicum*. After 24 h of stimulation, we extracted total RNA from LX-2 cells and assessed the expression of *α-SMA*, *Col 1 (α1)* and *Col 3 (α1)* (25, 26), and mRNA expression levels of these genes were upregulated, suggesting that egg-derived exosomes of *S. japonicum* may activate HSCs as well as promote ECM deposition (**Figure 1C**). Based on these results, we speculate that egg-derived exosomes of *S. japonicum* may expedite the progression of liver fibrosis in the host.

### *S. japonicum* Egg Exosome-Derived miRNA Mimics Can Activate LX-2

**Figure 2:**
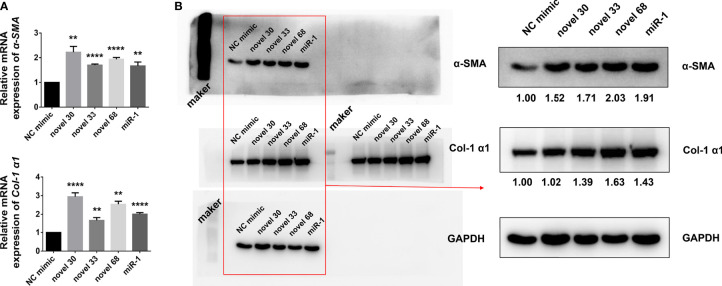
Novel miRNA-33 mimics can activate HSCs (human hepatic stellate cells). **(A)** LX-2 cells were transfected with several novel miRNA mimics (novel miRNA-30, novel miRNA-33, novel miRNA-68, miR-1, miRNA-NC), cultivated for 24 h, and mRNA levels of *α-SMA* and *Col 1 (α1)* were measured by qPCR. **(B)** LX-2 cells were transfected with several novel miRNA mimics (novel miRNA-30, novel miRNA-33, novel miRNA-68, miR-1, miRNA-NC), cultivated for 48 h, and expression levels of α-SMA and Col 1 (α1) were measured by western blotting. Results were compared with those of the NC mimic group. The abundances of α-SMA and Col 1 (α1) proteins were normalized against GAPDH. Results are averaged from three independent experiments, and Student’s *t*-tests were applied to assess differences between two groups (**p < 0.001, ****p < 0.0001).

Given that egg-derived exosomes of *S. japonicum* activated HSCs, hence we subjected the egg-derived exosomes of *S. japonicum* to high-throughput sequencing (27), and obtained a number of miRNAs (**Table 1**). We used these three new miRNA mimics to transfect LX-2 HSCs, and 24 h after transfection cells were collected and total RNA was extracted. After reverse transcription, the mRNA expression levels of *α-SMA* and *Col 1 (α1)* were measured by qPCR (**Figure 2A**), and the abundances of α-SMA and Col 1 (α1) proteins were measured by western blotting (**Figure 2B**). And the expression of collagen type III has no significant change at the protein level, compared with the NC group, and the Western blot image of collagen type III will be shown in **Supplementary Figure S1**. The results suggest that egg-derived exosomes of *S. japonicum* could activate LX-2. Through previous studies (28), we know that miRNAs can also regulate gene expression in a cross-species manner. Our results suggest that the novel miRNA-33 mimics from *S. japonicum* egg-derived exosomes could activate human HSCs through cross-species regulation (29).

### MiRNA Mimics From Egg-Derived Exosomes of *S. japonicum* Can Cause Liver Fibrosis in Mice

**Figure 3:**
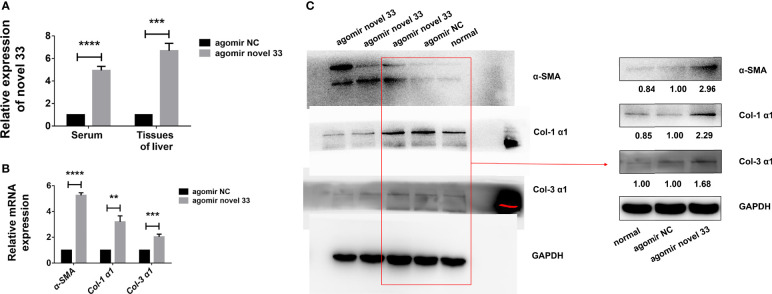
Novel miRNA-33 agomir causes liver fibrosis in mice. Six-week-old female C57BL/6 mice were injected with agomir NC (Ribo), agomir novel 33 (Ribo), or normal saline once a week for 6 weeks. Results were compared with the agomir NC group; the normal group was only used as a reference without comparative analysis. Only the gray values of the agomir NC group and the agomir novel 33 group were statistically analyzed, the blank group was not analyzed. **(A)** Novel miRNA-33 levels in mouse liver and serum were increased significantly. **(B)** qPCR results showing upregulation of *α-SMA, Col 1 (α1)*, and *Col 3 (α1)* at the mRNA level. **(C)** Western blotting results showing upregulation of α-SMA, Col 1 (α1), and Col 3 (α1) at the protein level. The abundances of α-SMA, Col 1 (α1), and Col 3 (α1) proteins were normalized against GAPDH, and miRNA expression levels were normalized against U6. Results are averaged from three independent experiments, and Student’s *t*-tests were applied to assess differences between two groups (**p < 0.001, ***p < 0.0005, ****p < 0.0001).

Since miRNA mimics from egg-derived exosomes of *S. japonicum* could activate human HSCs *in vitro*, we conducted experiments on a mouse model (female WT C57BL/6 mice not infected with *S. japonicum* cercariae) to verify the role of the novel miRNA-33 *in vivo*. As mentioned above, after establishing a mouse fibrosis model using miRNA agomir administered *via* the tail vein as described above, we found the degree of fibrosis was significant after mice were injected for 6 weeks with the novel miRNA mimics. Analysis of serum and liver tissues from mice injected with the novel miRNA-33 mimics for 6 weeks showed that the miRNAs in both samples were significantly upregulated (**Figure 3A**), indicating that the novel miRNA-33 can be processed and matured in mice.

In order to verify whether the novel miRNA-33 agomir could promote mouse liver fibrosis as expected, we performed qPCR experiments on liver tissues of fibrotic mice induced by miRNA agomir, and the results showed that *α-SMA, Col 1 (α1)*, and *Col 3 (α1)* were upregulated at mRNA level (**Figure 3B**). Moreover, the results of western blotting experiments on liver tissues of fibrotic mice induced by miRNA agomir showed that α-SMA, Col 1 (α1), and Col 3 (α1) were upregulated at the protein levels (**Figure 3C**), indicating that liver fibrosis was promoted in mice injected with novel miRNA-33 agomir. This suggests that our novel miRNA-33 agomir can be processed and matured in mice, and that it plays a role in promoting liver fibrosis in hosts.

### An miRNA Inhibitor Blunts Liver Fibrosis in Murine Schistosomiasis

**Figure 4:**
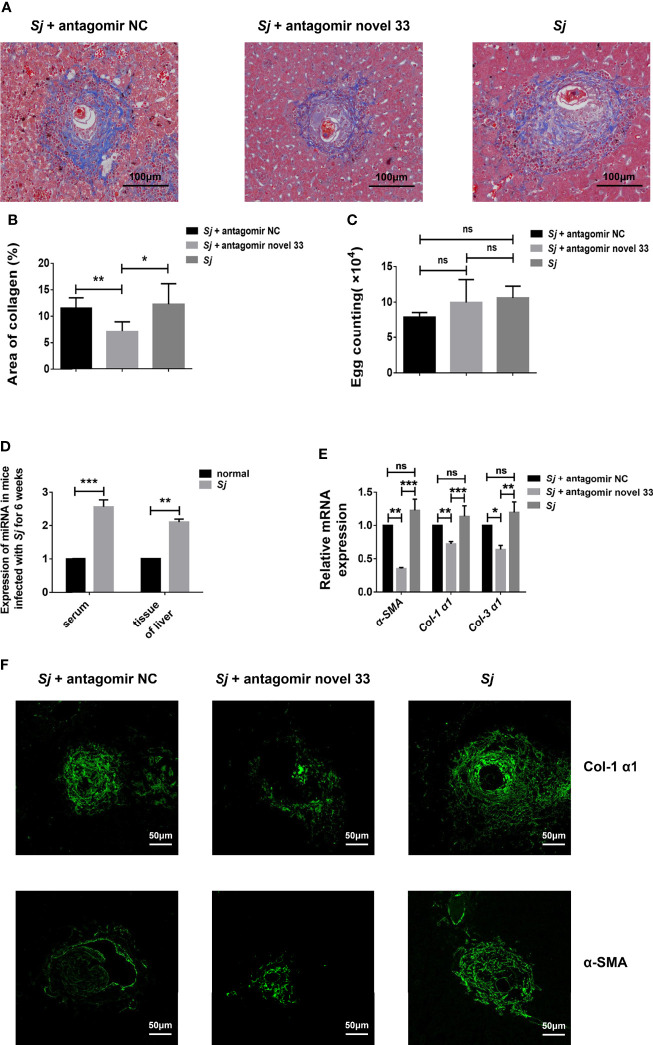
Inhibition of novel miRNA-33 reduces the degree of liver fibrosis. Six-week-old female C57BL/6 mice were percutaneously exposed to 20 ± 1 *S. japonicum* cercariae, and 120 μL of 20 nM miRNA antagomir NC(Ribo), miRNA antagomir novel 33 (Ribo), or normal saline were injected *via* the tail vein 7 days after mice were infected with *S. japonicum* cercariae, once a week for 6 weeks. The magnification of Masson-stained images is 10 ×, and the magnification of immunofluorescence image is 20 ×. **(A, B)** Masson staining showing that areas of collagen were decreased after treatment with novel miRNA-33 antagomir. **(C)** Egg count in mouse liver tissue showing there was no statistical difference between the three groups. **(D)** Expression of novel miRNA-33 in mice infected with *S. japonicum* for 6 weeks. **(E)** qPCR results showing downregulation of α-SMA, Col 1 (α1), and Col 3 (α1) at the mRNA level. **(F)** Immunofluorescence images showing downregulation of type I collagen and α-SMA after treatment with antagomir. mRNA expression levels of *α-SMA*, *Col 1 (α1)*, and *Col 3 (α1)* were normalized agaisnt *GAPDH*, and expression of novel miRNA-33 was normalized against U6. Results are averaged from three independent experiments, Student’s *t*-tests were applied to assess differences between two groups, and one-way analysis of variance (ANOVA) followed by Tukey’s post-tests was used for three or more groups (fluorescence is SpGreen; n = 20; *p < 0.05, **p < 0.001, ***p < 0.0005; NS, not significant).

After revealing that the novel miRNA-33 agomir could promote liver fibrosis in WT C57BL/6 female mice, we predicted that inhibiting this molecule may have therapeutic potential. After reviewing the relevant data, we treated mice infected with *S. japonicum* with a novel miRNA-33 antagonist (30). Masson staining showed that the collagen area around liver granulomas in the *S. japonicum* infection plus antagomir group was decreased compared with the *S. japonicum* infection plus saline groups and the *S. japonicum* infection plus antagomir NC groups (**Figures 4A, B**), while the number of *S. japonicum* eggs was not statistically different between the three groups (**Figure 4C**). Additionally, the results showed that after 6 weeks of infection with *S. japonicum*, the content of miRNA-33 in serum and liver tissue of mice were significantly increased compared with the normal group (**Figure 4D**). Furthermore, qPCR results showed that expression of *α-SMA*, *Col 1 (α1)*, and *Col 3 (α1)* was downregulated at the mRNA levels when the antagomir novel 33 group compared with the antagomir NC group (**Figure 4E**). Meanwhile, immunofluorescence analysis showed that expression of α-SMA and Col 1 (α1) was downregulated in the antagomir novel 33 group compared with the antagomir NC group (**Figure 4F**). While, the immunofluorescence images of type III collagen showed no significant changes in the antagomir novel 33 group compared with the antagomir NC group (**Supplementary Figure S2**). Our results suggest that the degree of fibrosis in mice infected with *S. japonicum* can be reduced by applying antagomir.

### TGF-β RI Is a Target Gene of Novel miRNA-33

**Figure 5:**
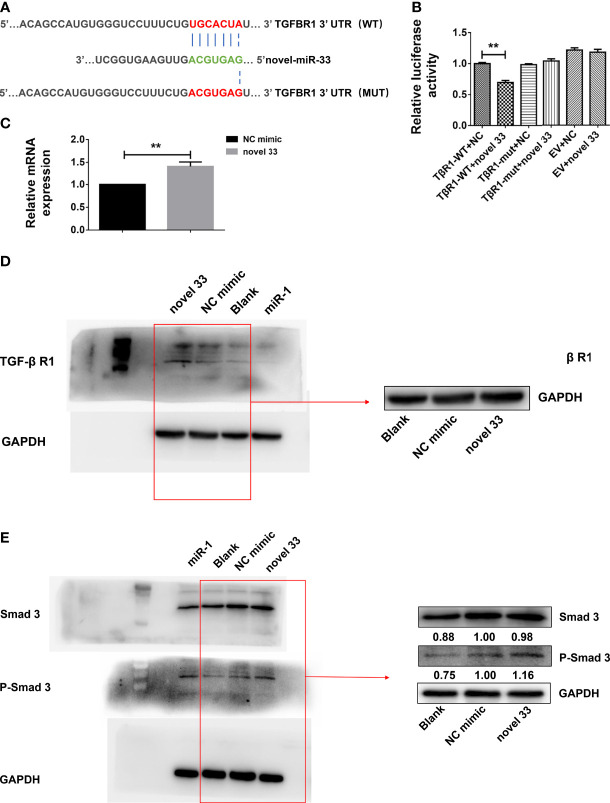
Novel miRNA-33 acts *via* the TGF β/Smad pathway to promote liver fibrosis. LX-2 cells were transfected with the novel miRNA-33 mimic and NC mimic. Total RNA was extracted 24 h after transfection of LX-2 cells, and protein was extracted 48 h after transfection of LX-2 cells. The blank control group did not receive treatment. Only the gray values of the NC mimic group and the novel 33 group were statistically analyzed; the blank group was not analyzed. Results were compared with the NC mimic group; the blank group was only used as a reference without comparative analysis. **(A)** Sequence alignment of novel miRNA-33 and the target region in the 3’UTR of TGF-β RI. **(B)** Luciferase reporter assays were performed on 293T cells transfected with pmirGLO-TGF-β RI 3’UTR-WT or pmirGLO-TGF-β RI-3’UTR-Mut in the absence or presence of novel miRNA-33 mimic. **(C)** qPCR results showing that novel miRNA-33 upregulates TGF-β RI expression. **(D)** Western blotting results showing that novel miRNA-33 upregulates TGF-β RI expression. **(E)** Smad3 is phosphorylated after transfection of LX-2 cells with novel miRNA-33. Expression levels of TGF-β RI, Smad3, and P-Smad3 were normalized against GAPDH. Results are averaged from three independent experiments, and Student’s *t*-tests were applied to assess differences between two groups **p < 0.001.

Online bioinformatics analysis using miRDB software was employed to predict target genes of novel miRNA-33. The results suggest that TGF-β RI may be a potential target gene of novel miRNA-33, and homology analysis showed that the seven base positions in the novel miRNA-33 sequence are complementary to bases of the TGF-β RI 3’UTR (**Figure 5A**). To determine whether novel miRNA-33 directly binds to this site, and to further validate the target genes of novel miRNA-33, we constructed complementary sites containing the WT 3’UTR of TGF-β RI (TGF-β RI 3’UTR-WT) and a mutant (TGF-β RI 3’UTR-MUT) and novel miRNA-33 using a dual luciferase gene reporter vector system. Dual-luciferase analysis of novel miRNA-33 mimics significantly reduced the activity of TGF-β RI 3’UTR luciferase, but had no effect on the activity of mutant reporter luciferase, while controls of the mimics had no effect on the activity of WT or mutant luciferase (**Figure 5B**). On the other hand, novel miRNA-33 inhibitors enhanced WT and mutant reporter luciferase activity. In addition, expression of TGF-β RI was upregulated at the mRNA levels after LX-2 cells were transfected for 24 h with the novel miRNA-33 mimics (**Figure 5C**), and TGF-β RI was upregulated at the protein levels after LX-2 cells were transfected for 48 h with the novel miRNA-33 mimics (**Figure 5D**) These results suggest an interaction between the novel miRNA-33 and the 3’UTR of TGF-β RI.

### Novel miRNA-33 Promotes Liver Fibrosis *via* the TGF-β/SMAD3 Signaling Pathway

Previous studies reported that mature TGF-β 1 binds to type I and type II cell surface receptor complexes (Tβ RI and Tβ RII), and each receptor complex consists of a small cysteine-rich extracellular region and an intracellular region mainly composed of a kinase domain (31, 32). In TGF-β/Smad signaling, TGF-β I binding to TGF-β RII initiates intracellular signaling, and TGF-β I then activates TGF-β RI kinase, resulting in Smad2 and Smad3 phosphorylation (33). Subsequently, activated Smad2 and Smad3 form oligomeric complexes with Smad4, and these oligomeric complexes translocate to the nucleus, where they regulate the transcription of target genes (34). TGF-β intracellular signals are transmitted by phosphorylation of Smad2 and Smad3 (34, 35). Moreover, we also know that expression of many fibroblast genes (collagen) and markers such as α-SMA depends on Smad3, and Smad3 directly binds to DNA sequences that regulate these target genes, thereby regulating the process of liver fibrosis (36). From our results (**Figure 5E**), we know that novel miRNA-33 can upregulate TGF-β RI, which is conducive to the phosphorylation of Smad3 and the promotion of fibrosis in LX-2 cells. The results of qPCR and western blotting showed that TGF-β RI in novel miRNA-33 transfection group was significantly upregulated at both mRNA and protein levels, compared with the control group. Type I and II cell surface receptor complexes can activate the phosphorylation of Smad3, which enter the nucleus and bind to nuclear collagen genes, thereby inducing the overexpression of collagen and promoting fibrosis (34).

## Discussion

This study revealed that novel miRNA-33 is related to the occurrence and progression of liver fibrosis during *S. japonicum* infection. Specifically, (a) a large number of novel miRNA-33 molecules from egg-derived exosomes of *S. japonicum* are present in the liver and serum of hosts infected with *S. japonicum*, (b) the novel miRNA-33 participates in the activation of HSCs and the occurrence of liver fibrosis in the host, (c) the novel miRNA-33 from *S. japonicum* regulates post-transcriptional processes in the host in a cross-species manner, (d) the novel miRNA-33 promotes liver fibrosis through the TGF-β/Smad3 signaling pathway.

In view of previous studies, we found that novel miRNA-33 from *S. japonicum* is present in both the serum and liver tissue of the host, suggesting that it may be a new marker of liver fibrosis. Additionally, it activates LX-2 cells, causing them to overexpress and deposit specific molecules in the ECM, resulting in host liver fibrosis, consistent with previous research (37). The results of the present study suggest that novel miRNA-33 from *S. japonicum* can regulate the post-transcriptional process of the host in a cross-species manner (29, 38, 39). From our experimental data, we know that the novel miRNA-33 agomir can cause fibrosis in mice. After treatment with novel miRNA-33 antagomir, the degree of liver fibrosis in mice was decreased significantly, which suggests that novel miRNA-33 is a new biomarker of schistosomiasis that could serve as a target for the treatment of liver fibrosis caused by *S. japonicum*.

Consistent with previous research, our study suggests that miRNAs perform various roles in post-transcriptional regulation in hosts (40). Based on the dual-luciferase reporter system, we found that novel miRNA-33 has complementary pairing sequences with target genes, and could reduce luciferase activity of target genes. However, we believe that this is not reflective of the role of the miRNA in the degradation of target genes. On the contrary, we speculate that novel miRNA-33 may enhance the stability of the target gene and/or enhance the stability of its translation process by specifically binding to the target gene. Evidence suggests that miRNAs not only play a role in stabilizing and repairing virus mRNAs during virus protein expression, but also upregulate host mRNA transcription and translation in eukaryotes, therefore, we propose that miRNAs may also enhance the translation stability of host mRNAs in the process of cross-species regulation, thereby upregulating target gene translation products (41). Consequently, our results imply that novel miRNA-33 upregulates the expression of host target genes in a cross-species manner (42).

From previous studies, we know that after activation, mature TGF-β 1 binds to type I and type II cell surface receptor complexes. Each receptor complex consists of a small extracellular domain rich in cysteine residues and an intracellular region mainly composed of a kinase domain. In TGF-β/Smad signal transduction, TGF-β 1 initiates intracellular signal transduction by binding to TGF-β RII and TGF-β 1 then activates TGF-β RI kinase, leading to Smad2 and Smad3 phosphorylation (31, 32).The potential mechanism of exosomes remains unknown and this is a barrier to the development of effective treatments. Our findings confirm that a novel miRNA from egg-derived exosomes of *S. japonicum* can regulate the fibrosis process in the host in a cross-species manner. This knowledge may assist the establishment of a method for treating hepatic fibrosis caused by *S. japonicum*.

## Data Availability Statement

The original contributions presented in the study are included in the article/**Supplementary Material**. Further inquiries can be directed to the corresponding authors.

## Ethics Statement

The animal study was reviewed and approved by the Laboratory Animal Welfare & Ethics Committee (LAWEC) of the National Institute of Parasitic Diseases, Chinese Center for Disease Control and Prevention (Chinese Center for Tropical Diseases Research).

## Author Contributions

YW and JC conceived and designed the experiments. YW, WG, HZ, YH, LW, and YS performed the experiments. YW, WG, JC, and GY analyzed the data. JC, GY, and WG contributed reagents and materials. YW, JC, and WG wrote and revised the manuscript. All authors contributed to the article and approved the submitted version.

## Funding

This work was supported by the National Nature Science Foundation of China (Nos. 81772225 and 81971969 to JC; No.81802032 to WG), and the Three-Year Public Health Action Plan (2020-2022) of Shanghai (No. GWV-10.1-XK13 to JC). The funders had no role in the study design, data collection, and analysis, decision to publish, or preparation of the manuscript.

## Conflict of Interest

The authors declare that the research was conducted in the absence of any commercial or financial relationships that could be construed as a potential conflict of interest.

## Publisher’s Note

All claims expressed in this article are solely those of the authors and do not necessarily represent those of their affiliated organizations, or those of the publisher, the editors and the reviewers. Any product that may be evaluated in this article, or claim that may be made by its manufacturer, is not guaranteed or endorsed by the publisher.

## Supplementary Material

Supplementary Data Sheet 1 Excel spreadsheet containing, in separate sheets, the numerical data for **Figures 1C**, **2A**, **3A, B**, **4B–E** and **5B, C**.

Supplementary Data Sheet 2 Sourced data for the immunoblotting, TEM, masson staining, immunofluorescence, and sequence alignment are shown in ** Figures 1A, B**, **2B**, **3C**, **4A, F, 5A**, **D, E**, **Supplementary Figures S1**, and **S2**.
