# Supplementary material for: A Novel miRNA From Egg-Derived Exosomes of Schistosoma japonicum Promotes Liver Fibrosis in Murine Schistosomiasis
Source: Front Immunol. 2022 Apr 27;13:860807. doi: 10.3389/fimmu.2022.860807 (PMC9094574; doi:10.3389/fimmu.2022.860807)
Supplement: Supplementary Data Sheet 1 — Excel spreadsheet containing, in separate sheets, the numerical data for Figures 1C, 2A, 3A, B, 4B–E and 5B, C. [file DataSheet_1.pdf]

Supplementary Material

Supplementary Figures

Supplementary Figure

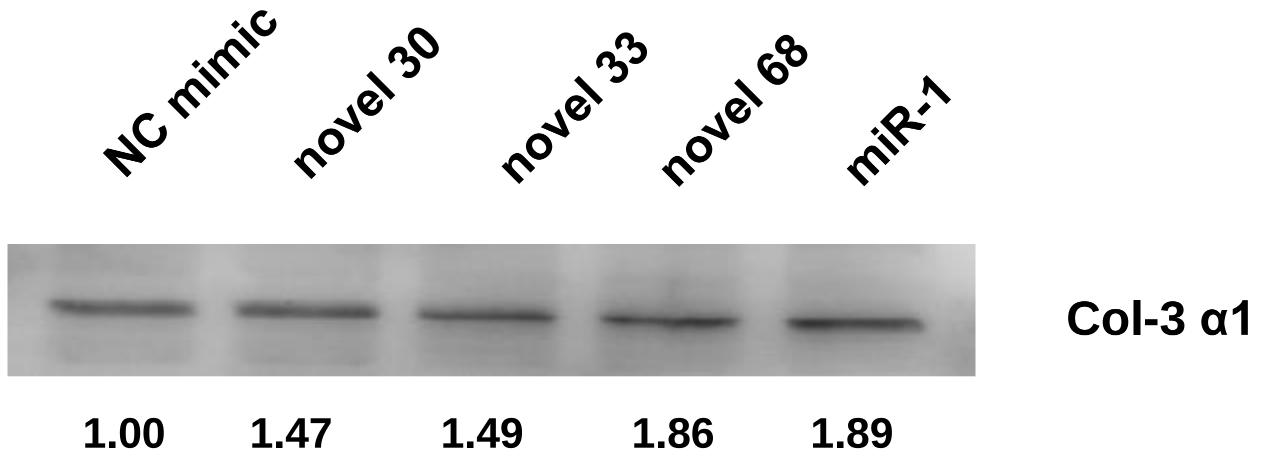

Supplementary Figure S1. Novel miRNA-33 can upregulate the protein expression of Col 3 (α1) in LX-2 cells. LX-2 cells were transfected for 48 hours with several novel miRNA mimics (novel miRNA-30, novel miRNA-33, novel miRNA-68, miR-1, and miRNA-NC), and the expression of Col 3 (α1) was determined by western blotting. Results of novel miRNA-30, novel miRNA-33, novel miRNA-68, and miR-1 group were compared with those of the miRNA-NC group. The abundances of Col 3 (α1) proteins were normalized against GAPDH.

Supplementary Figure

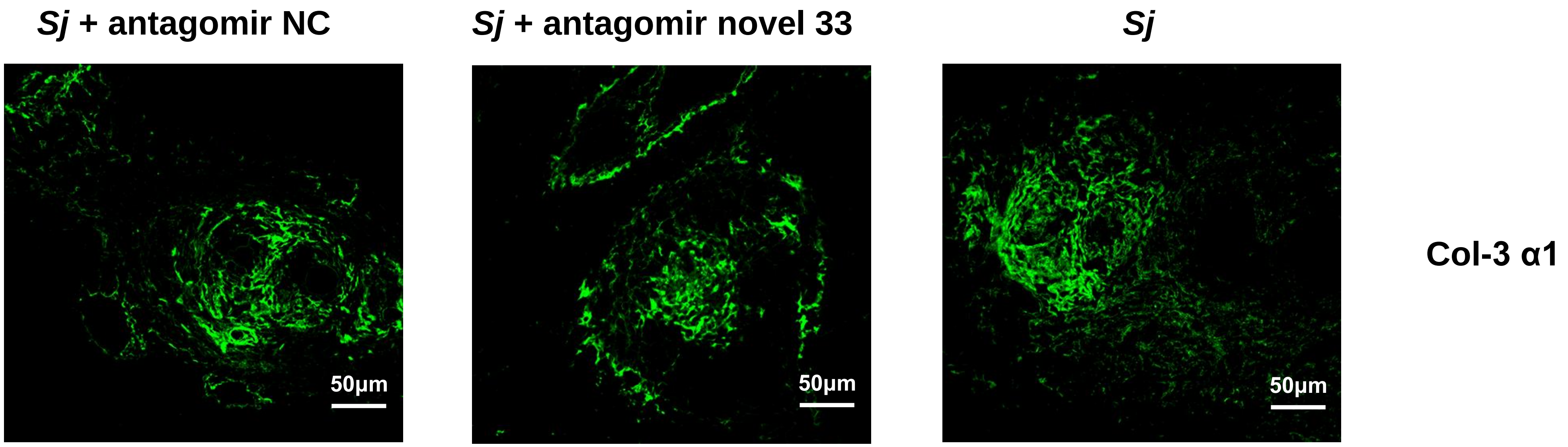

Supplementary Figure S2. The expression of type III collagen did not change significantly after treatment with a novel miRNA-33 antagomir. Six-week-old female C57BL/6 mice were percutaneously exposed to 20 ± 1 *S. japonicum* cercariae, and 120 μL of 20 nM miRNA antagomir NC(Ribo), miRNA antagomir novel 33 (Ribo), or normal saline were injected via the tail vein 7 days after mice were infected with *S. japonicum* cercariae, once a week for 6 weeks. The magnification of immunofluorescence image is 20 ×.
